# Evaluation of performance of two different chest tubes with either a sharp or a blunt tip for thoracostomy in 100 human cadavers

**DOI:** 10.1186/1757-7241-20-10

**Published:** 2012-02-02

**Authors:** Clemens M Ortner, Kurt Ruetzler, Nikolaus Schaumann, Veit Lorenz, Peter Schellongowski, Ernst Schuster, Ramez M Salem, Michael Frass

**Affiliations:** 1 University of Washington, Department of Anesthesiology and Pain Medicine, 1811 East Lynn Street, Seattle, 98112 WA, USA; 2 Medical University of Vienna, Department of Cardiothoracic and Vascular Anesthesia and Intensive Care Medicine, Waehringer Guertel 18-20, 1090 Vienna, Austria; Outcomes Research Consortium; 3 Medical University Vienna, Department of Internal Medicine I, Intensive Care Unit, Waehringer Guertel 18-20, 1090 Vienna, Austria; 4 Medical University Vienna, Core Unit for Medical Statistics and Informatics, Waehringer Guertel 18-20, 1090 Vienna, Austria; 5 Advocate Illinois Masonic Medical Center Chicago, Department of Anesthesiology, 836 W. Wellington Avenue, Chicago, Illinois 60657, USA; 6 AUVA Hospital Meidling, Kundratstrasse 37, 1120 Vienna, Austria; 7 Wilhelminenspital, Department of Dermatology, Montleartstrasse 37, 1160 Vienna, Austria

**Keywords:** chest tubes, thoracostomy, cadaver, pneumothorax, hemothorax, pleural effusion, empyema

## Abstract

**Background:**

Emergent placement of a chest tube is a potentially life-saving procedure, but rate of misplacement and organ injury is up to 30%. In principle, chest tube insertion can be performed by using Trocar or Non-trocar techniques. If using trocar technique, two different chest tubes (equipped with sharp or blunt tip) are currently commercially available. This study was performed to detect any difference with respect to time until tube insertion, to success and to misplacement rate.

**Methods:**

Twenty emergency physicians performed five tube thoracostomies using both blunt and sharp tipped tube kits in 100 fresh human cadavers (100 thoracostomies with each kit). Time until tube insertion served as primary outcome. Complications and success rate were examined by pathological dissection and served as further outcomes parameters.

**Results:**

Difference in mean time until tube insertion (63s vs. 59s) was statistically not significant. In both groups, time for insertion decreased from the 1^st ^to the 5^th ^attempt and showed dependency on the cadaver's BMI and on the individual physician. Success rate differed between both groups (92% using blunt vs. 86% using sharp tipped kits) and injuries and misplacements occurred significantly more frequently using chest tubes with sharp tips (p = 0.04).

**Conclusion:**

Data suggest that chest drain insertion with trocars is associated with a 6-14% operator-related complication rate. No difference in average time could be found. However, misplacements and organ injuries occurred more frequently using sharp tips. Consequently, if using a trocar technique, the use of blunt tipped kits is recommended.

## Background

Pneumothorax occurs in 5-41% of all thoracic injuries [1-3], and in up to 25% of patients suffering multiple injuries [4,5]. Besides hemothorax, pneumothorax is the most frequent indication for insertion of a chest tube in trauma patients [4,5]. Complications like tube malposition have been reported in up to 25% of attempted insertions of a chest tube [1,6-8]. In an emergency setting, needle decompression is a widely used technique to manage a tension pneumothorax. However, this is an inexact and potentially dangerous technique. It may be ineffective, and requires subsequent chest tube insertion in a significant number of cases [9-13]. Therefore, needle decompression may be considered primarily as a diagnostic manoeuvre. In advanced pre-hospital emergency care, tube thoracostomy serves as the gold standard in treating tension pneumothorax [14]. The success rate of chest tube placement in a pre-hospital emergency setting ranges from 79 to 95% [9].

There are two possible approaches available for chest tube placement: the ventral approach (2^nd^-3^rd ^Intercostal space in the mid-clavicular line, according to Monaldi); and the lateral approach (4^th^-6^th ^intercostal space in the mid-axillary line, according to Bülau) [15,16]. The lateral is considered optimal in trauma patients [17,18].

One of two basic techniques to insert a chest tube is usually applied: the trocar or the non-trocar technique. The trocar technique alleviates guidance as compared to non-trocar techniques, but has the potential of increased complication rates [17,18]. However, a recently described technique using the fingers for exploring the pleural space and simultaneously guiding the trocar improves safety and has shown equally low complication rates as with the non-trocar techniques [19].

If using a trocar technique, two main types of chest tube devices are currently available in order to perform thoracic drainage: one equipped with a sharp tip and the other with a blunt tip. However, it is not known how these different trocar tips differ in terms of safe, effective and efficient chest tube insertion. Thus, the aim of this study was to test the hypothesis that there is no difference between blunt and sharp tip devices with respect to success rate of placement, complications, and time to accomplish the procedure.

## Methods

With approval of the Local Ethical Committee of the Medical University of Vienna and after having obtained informed consent, twenty emergency medicine residents between their 2^nd ^and 5^th ^year of residency participated in this controlled, randomised, single-center study. Only two of them had previously performed one tube thoracostomy; the other 18 physicians had no previous hands-on training or experience in performing any tube thoracostomy.

All physicians attended a one-hour-long standardised lecture covering relevant aspects of basic anatomic, physiologic and pathological principles, clinical indications and contraindications, as well as complications of insertion of chest tubes. Following the lecture, emergency physicians participated in a practical demonstration, where insertion of chest tubes into a human cadaver using the two different devices was demonstrated by an experienced physician.

After the lecture and practical demonstration, each physician had to perform the procedure on one side of an adult human cadaver using a tube kit selected in a computer-generated randomised sequence. Afterwards, the physician had to perform the procedure using the other kit on the other side of chest. This procedure was repeated monthly for 5 consecutive months by each physician in order to evaluate a possible training or learning effect. In this manner, 20 different physicians inserted a total of 100 chest tubes with each kit. Physicians were not allowed to watch each other to avoid any observational teaching bias or learning effect.

**Figure 1:**
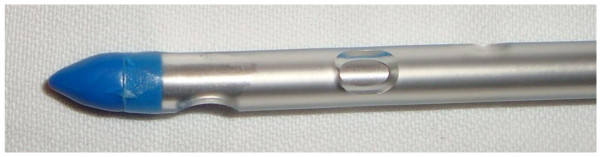
**Tyco^® ^tube thoracostomy unit with a blunt tip (Tyco^® ^Thoracic Trocar and drain, Athlone, Ireland)**.

Chest tube insertion was performed with the following two kits:

Kit 1: The Tyco^® ^tube thoracostomy unit (Tyco^® ^Thoracic Trocar and drain, Athlone, Ireland) for the aseptic introduction of a chest tube equipped with a blunt tip consisting of a thoracic trocar 24 F (8.0 mm × 332 mm) inside of a drain [Figure 1].

**Figure 2:**
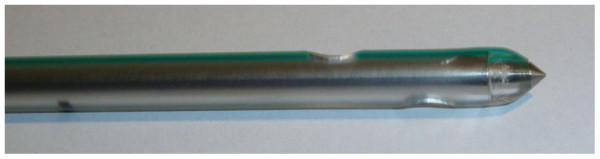
**Vygon^® ^tube thoracostomy unit (Thoracic Trocar and drain, Vygon^®^, Norristown, Philadelphia) with sharp tip**.

Kit 2: The Vygon^® ^tube thoracostomy unit (Thoracic Trocar and drain, Vygon^®^, Norristown, Philadelphia) for the aseptic introduction of a chest tube equipped with a sharp tip consisting of a thoracic trocar 24 F (8.0 mm × 280 mm) inside of a drain [Figure 2].

With both kits the following thoracostomy insertion technique was applied, as recently described as an alternative and safe technique in closed tube thoracostomy by Dural [19].

1. A 2 cm incision through the skin and subcutaneous tissue just superior and parallel to the caudal rib of the fourth or fifth intercostal space in the mid-axillary line is performed.

2. The index finger palpates and widens the incision.

3. The chest tube is guided bluntly with the index finger through the chest wall and advanced over the trocar into the pleural space.

The physicians were allowed only one attempt per kit and cadaver. In total, ten chest tube placement attempts were documented for each emergency physician (5 attempts using kit 1 and 5 attempts using kit 2). After chest tube placement, all human cadavers underwent post-mortem examination by an independent pathologist not involved in this study. The pathologist inspected the cadavers for accuracy of placement and complications, including damage to internal organs and lacerations to the lung, liver, spleen, or diaphragm, or injuries to major extra thoracic viscera.

Age, sex, height, weight, body mass index (BMI) of the cadavers, and the intercostal puncture site were recorded. BMI > 25 kg/m^2 ^was defined as obesity. The primary outcome parameter was efficiency of tube insertion as defined by the time to complete the procedure (time from skin incision to successful tube insertion). Secondary outcome parameters were learning effect (= difference in time of the procedure of first try versus the fifth try), the incidence of complications (= injuries to internal organs) and its association with BMI, the performing physician and the specific attempt.

100 adult human cadavers, undergoing obligatory post-mortem examination, were used for this study. This sample size was judged acceptable and was based on what was considered achievable and realistic within a reasonable time frame. Human cadavers were refrigerated and non-formalin-fixed within 4-24 hours after death. All human cadavers, except human cadavers with chest or abdominal trauma, obvious chest pathology, or any form of infection (tuberculosis, hepatitis C, or HIV) were used

### Statistical analysis

For the statistical description of the cadavers (demographical data) means and standard deviations (SD) were calculated. For the primary end-point (total time of procedure) we performed paired t-tests for each trial individually as well as a multifactorial analysis of variance (trial number, BMI of the cadaver, individual physician). For the secondary end-points (complications, failures, categorised BMI of cadaver) a contingency table was built and analysed by the chi-square test or by Fisher's exact test respectively. SAS, Version 9.1, Cary, NC, was used. The null hypothesis was rejected when the two sided significance level was below 5%.

## Results

Both tube types were inserted into the same cadavers per physician per day; therefore demographic characteristics are equal in both groups [table 1]. Two trials with kit 1 had to be aborted because of a material defect and were excluded from analysis. Results from all other 98 thoracic tube placements were included in the analysis.

**Table 1:** demographical data of human cadavers

	Mean ± SD	**Min**.	**Max**.
Age (years)	63.56 ± 13.41	24	87
		
Sex			
male	66		
female	34		
		
Height (cm)	171.54 ± 9.13	154	196
Weight (kg)	82.96 ± 21.85	39	150
Body mass index (kg/m^2^)	28.09 ± 6.65	15.23	46.92

Data are presented as Mean ± Standard Deviation (SD) respectively as well as absolute values

Mean time for chest tubes insertion with blunt tips was 63 seconds (± 25), versus 59 seconds (± 22) using sharp tips (p = 0.41).

Furthermore, no difference in mean time comparing each trial, from first to fifth trial, could be shown between kits. However, mean time for insertion decreased equally for both kits from first to fifth attempt [18 ± 39 s (p = 0.05) with kit 1; and 20 ± 20 s (p = 0.003) using kit 2] [table 2].

**Table 2:** Time for Chest Tube Insertion for each Trial

	Blunt Tip	Sharp Tip	p-value
Trial 1	70 (45)	70 (32)	0.98
Trial 2	61 (27)	64 (34)	0.54
Trial 3	76 (64)	60 (35)	0.33
Trial 4	57 (27)	50 (22)	0.23
Trial 5	52 (23)	50 (25)	0.48
Trial 1 - 5	63 (25)	59 (22)	0.57

Mean time (Standard Deviation) until chest tube placement in cadaver for 20 physicians, using tube set with blunt or sharp tip

Post-mortem examination revealed that chest tubes were accurately placed into the pleural space in 92 out of 98 (94%) cadavers using kit 1, and in 86 out of 100 (86%) using kit 2 [table 3]. No difference between right and left sided injuries or misplacement could be found.

**Table 3:** Summary of correct position respectively misplacements/injuries

	Blunt Tip	Sharp Tip
n	100	100
Interpleural Space	92	86
Subphrenical Misplacement	2	4
Extra thoracic Misplacement	2	5
Liver Injury	2	1
Spleen Injury	0	4
Aborted	2	0

Results are expressed as absolute values

Injuries and misplacements occurred more frequently using chest tubes with kit 2, which contains a sharp tipped trocar (p = 0.04). Injuries to internal organs occurred when the tube was inserted via the seventh or eighth intercostal space, based on incorrect application by the operators. Two trials using kit 1 had to be aborted due to damage of the thoracic drain by the trocar, resulting in inability to introduce the chest tube. In both groups no lung or heart lacerations or stomach injuries were observed. Contingency table calculation demonstrated an association of organ injuries with the use of kit 2 (p = 0.043). The same 2 operators misplaced the chest tube using kit 2 during all attempts, resulting in misplacement due to the operators (p = 0.001), (kit1: p = 0.62). In contrast, no association could be found between incidence of misplacement and BMI of the cadaver (kit1: p = 0.62; kit 2: p = 0.28), or the number of prior attempts. Errors appeared to occur at any point in the training of particular physicians.

In the analysis of variance with three factors (including BMI, performing physician and number of trials), it could be shown that the time for insertion was dependent on the cadaver's BMI (kit 1: *p *< 0.002, kit 2: *p *< 0.03) and on the individual physician (p < 0.001 in both groups).

## Discussion

To our knowledge, this is the first published controlled randomised study evaluating efficiency, training effect, and safety of tube thoracostomy, comparing kits equipped with either blunt or sharp tips in a sample of human cadavers.

Chest thoracostomy using sharp tipped trocars was around 4 seconds faster than using blunt tips; however, this difference seems to be clinically not relevant. Insertion times were increased in obese cadavers, operator related, and could be reduced with training. Using either kit led to high complication rates, with a significantly increased incidence of organ injuries using sharp tipped as compared to blunt tipped trocars.

In 2009, the National Patient Safety Agency in the United Kingdom reported that equipment problems, adequate training, and site selection are crucial factors influencing effective and safe chest drain insertion [20]. Pleural drainage techniques are not uncomplicated, and have the potential to cause life-threatening injury [14]. Even though there is ongoing debate about its safety, the sharp tipped chest trocar technique is still widely used [8,21,22]. A recent study by *Dural et al*. reports higher success rates without difference in complication rate using a sharp tipped trocar when compared to a surgical technique in human patients [19]. In this study, the usual trocar technique was modified such that the pleural space and adhesions were bluntly dissected with a finger before advancing the thoracic trocar and drain. In other words, the trocar was not used to dissect, but to guide the drain into the pleural space. This technique is comparable to the one described in this report. Rates of misplacement or ineffective drainage for these trained cardiothoracic surgeons were reported to be 13.3% with the surgical technique and 7.8% with the trocar technique [19]. The authors used a kit similar to kit 2 described in this study, which in our training regime resulted in a high complication rate of 14% overall. Nevertheless, although our complication rate was higher than that reported by *Dural et al*., it is perhaps not surprising if one considers the relative lack of experience of the physicians that participated in our study, compared to that of trained surgeons that participated in the study of Dural. However, the Early Management of Severe Trauma (EMST)/Advanced Trauma Life Support (ATLS) courses and current guidelines still advocate the non-trocar technique as a safer method of chest tube insertion. The comparable high complication rates observed in our study consequently do not give an indication to change that policy and refute the findings by *Dural et al *claiming the trocar technique being equally safe.

Although smaller tubes are (< 14 F) increasingly used [23], we evaluated the use of medium sized bore tubes (24 F), as they are recommended for managing hematothorax, mechanical ventilation barotrauma, and some cases of pneumothorax [24].

In our study, surprisingly, no relevant difference in thoracostomy insertion time between kits could be demonstrated with a sample size of 100 trials in each group. Our sample size was set randomly, without a pilot study to establish the power of this study. By increasing its sample size the probability to reject the null hypothesis could have been increased. However, we believe that our results reliably demonstrate that there is no clinically relevant difference in insertion efficiency between the kits. Interestingly, from first to fifth attempt, time for insertion could be significantly reduced by 20 seconds, indicating a training effect. Using human cadavers as a teaching model, *Proano et al*. demonstrated significant reductions in insertion times [25]: Average time for first insertion attempt was 86 seconds, which decreased to 34 seconds at the fourth attempt. This strong training effect may be explained by the fact that trainees performed four procedures in a row in the same session. In our setting, by contrast, each thoracostomy was performed in one month intervals. So even though mean values slightly differ between studies, our results confirm a training effect and emphasise the usefulness of this training model.

In our study, insertion time was increased in cadavers with higher BMI, demonstrating that obesity impedes chest tube insertion. No association between increased BMI and tube malposition could be found, which is in accordance with findings reported in critically ill patients [22]. However, in both studies the correlation of BMI and chest tube malposition has been reported as a secondary outcome and was not sufficiently powered to draw a definite conclusion.

The use of human cadavers for chest tube insertion is a well-established method for training purposes [25]. There are, however, several limitations. The condition of the somatic tissue present in the cadaveric state is significantly different from that found in the living human. Furthermore, complications, such as bleeding of an intercostal artery, infections, and problems arising with chest tube removal cannot be simulated. Despite these limitations, we could demonstrate that misplacement or organ injury occurs significantly more frequently using sharp tipped trocars. We taught trainees to insert the chest tubes via the fourth or fifth intercostal space in the mid-axillary line because the diaphragm may rise to the level of the fourth intercostal space during full expiration. However, as described above, misplacements and organ injuries occurred mainly because chest tubes were placed via the seventh or eighth intercostal space. This confirms the conclusion by *Lamont et al*. that difficulties in identifying intercostal structures impede correct placement [20].

Interestingly, even though all participants had the same level of training, misplacement was operator related, and not related to number of prior training sessions. This shows that training duration may need to be customised to the individual trainee's learning rate. Cadaveric simulation may be an effective device to identify individuals that may benefit from additional training. Furthermore, it emphasises general recommendations that adequate training is a primary influence on the incidence of chest tube complications [26,27].

One might be surprised about the low level of experience in inserting chest drains of emergency physicians with at least 2 years of training. In our study center, the General Hospital of Vienna (AKH-Wien, Vienna, Austria), emergency care is divided into a medical emergency department and a trauma emergency department. Our physicians were recruited entirely from the medical department, as we believed training effects could be better demonstrated with physicians with little surgical training.

## Conclusion

To conclude, in this study, chest drain insertion with trocars is associated with a 6-14% complication rate that is operator related. The use of sharp tipped trocars increases the incidence of complications without facilitating the speed of the procedure. Therefore, sharp tipped trocars should not be used in routine clinical setting. Insertion time can be reduced with advanced training, however, is operator related and influenced by the patient's BMI.

## Competing interests

None of the authors has a personal or financial interest in this research. All materials were provided by department and university funding.

## Authors' contributions

CO made substantial contributions to analysis and interpretation of data, drafted the manuscript, revised it critically for important intellectual content, and has given final approval of the version to be published. KR made substantial contributions to analysis and interpretation of data, drafted the manuscript, revised it critically for important intellectual content, and has given final approval of the version to be published. NS, VL and PS contributed to the study design and acquisition of data. ES performed the statistical analysis, and RS analysed and interpreted data. MF contributed to study design and interpretation of data. All authors read and approved the final manuscript.

## References

[B1] Bailey RC Complications of tube thoracostomy in trauma J Accid Emerg Med 2000 17 111 4 10.1136/emj.17.2.111 10718232 PMC1725338

[B2] Demartines N Kiener A Scheidegger D Harder F Thoracic drainage at the accident site Helv Chir Acta 1990 57 273 7 2074186

[B3] Gaillard M Herve C Mandin L Raynaud P Mortality prognostic factors in chest injury J Trauma 1990 30 93 6 10.1097/00005373-199001000-00015 2296073

[B4] Heng K Bystrzycki A Fitzgerald M Gocentas R Bernard S Niggemeyer L Cooper DJ Kossmann T Complications of intercostal catheter insertion using EMST techniques for chest trauma ANZ J Surg 2004 74 420 3 10.1111/j.1445-1433.2004.03023.x 15191471

[B5] Di Bartolomeo S Sanson G Nardi G Scian F Michelutto V Lattuada L A population-based study on pneumothorax in severely traumatized patients J Trauma 2001 51 677 82 10.1097/00005373-200110000-00009 11586158

[B6] Etoch SW Bar-Natan MF Miller FB Richardson JD Tube thoracostomy. Factors related to complications Arch Surg 1995 130 521 5 discussion 5-6 10.1001/archsurg.1995.01430050071012 7748091

[B7] Eddy AC Luna GK Copass M Empyema thoracis in patients undergoing emergent closed tube thoracostomy for thoracic trauma Am J Surg 1989 157 494 7 10.1016/0002-9610(89)90643-0 2712206

[B8] Huber-Wagner S Korner M Ehrt A Kay MV Pfeifer KJ Mutschler W Kanz KG Emergency chest tube placement in trauma care - which approach is preferable? Resuscitation 2007 72 226 33 10.1016/j.resuscitation.2006.06.038 17141396

[B9] Waydhas C Sauerland S Pre-hospital pleural decompression and chest tube placement after blunt trauma: A systematic review Resuscitation 2007 72 11 25 10.1016/j.resuscitation.2006.06.025 17118508

[B10] Leigh-Smith S Harris T Tension pneumothorax-time for a re-think? Emerg Med J 2005 22 8 16 10.1136/emj.2003.010421 15611534 PMC1726546

[B11] Butler KL Best IM Weaver WL Bumpers HL Pulmonary artery injury and cardiac tamponade after needle decompression of a suspected tension pneumothorax J Trauma 2003 54 610 1 10.1097/01.TA.0000046380.92001.81 12634547

[B12] Rawlins R Brown KM Carr CS Cameron CR Life threatening haemorrhage after anterior needle aspiration of pneumothoraces. A role for lateral needle aspiration in emergency decompression of spontaneous pneumothorax Emerg Med J 2003 20 383 4 10.1136/emj.20.4.383 12835367 PMC1726134

[B13] Barton ED Epperson M Hoyt DB Fortlage D Rosen P Prehospital needle aspiration and tube thoracostomy in trauma victims: a six-year experience with aeromedical crews J Emerg Med 1995 13 155 63 10.1016/0736-4679(94)00135-9 7775785

[B14] Aylwin CJ Brohi K Davies GD Walsh MS Pre-hospital and in-hospital thoracostomy: indications and complications Ann R Coll Surg Engl 2008 90 54 7 18201502 10.1308/003588408X242286 PMC2216718

[B15] Tomlinson MA Treasure T Insertion of a chest drain: how to do it Br J Hosp Med 1997 58 248 52 9488797

[B16] Symbas PN Chest drainage tubes Surg Clin North Am 1989 69 41 6 2643182 10.1016/s0039-6109(16)44733-x

[B17] Fitzgerald M Mackenzie CF Marasco S Hoyle R Kossmann T Pleural decompression and drainage during trauma reception and resuscitation Injury 2008 39 9 20 10.1016/j.injury.2007.07.021 18164300

[B18] Laws D Neville E Duffy J BTS guidelines for the insertion of a chest drain Thorax 2003 58 Suppl 2 ii53 9 12728150 10.1136/thorax.58.suppl_2.ii53 PMC1766017

[B19] Dural K Gulbahar G Kocer B Sakinci U A novel and safe technique in closed tube thoracostomy J Cardiothorac Surg 2010 5 21 10.1186/1749-8090-5-21 20370923 PMC2867787

[B20] Lamont T Surkitt-Parr M Scarpello J Durand M Hooper C Maskell N Insertion of chest drains: summary of a safety report from the National Patient Safety Agency BMJ 2009 339 b4923 10.1136/bmj.b4923 19955139

[B21] Phua GC Wahidi MM ICU procedures of the critically ill Respirology 2009 14 1092 7 10.1111/j.1440-1843.2009.01643.x 19909459

[B22] Remerand F Luce V Badachi Y Lu Q Bouhemad B Rouby JJ Incidence of chest tube malposition in the critically ill: a prospective computed tomography study Anesthesiology 2007 106 1112 9 10.1097/01.anes.0000267594.80368.01 17525585

[B23] Fysh ET Smith NA Lee YC Optimal chest drain size: the rise of the small-bore pleural catheter Semin Respir Crit Care Med 2010 31 760 8 10.1055/s-0030-1269836 21213208

[B24] Light RW Pleural controversy: optimal chest tube size for drainage Respirology 2011 16 244 8 10.1111/j.1440-1843.2010.01913.x 21166742

[B25] Proano L Jagminas L Homan CS Reinert S Evaluation of a teaching laboratory using a cadaver model for tube thoracostomy(1) J Emerg Med 2002 23 89 95 10.1016/S0736-4679(02)00468-7 12217479

[B26] Ball CG Lord J Laupland KB Gmora S Mulloy RH Ng AK Schieman C Kirkpatrick AW Chest tube complications: how well are we training our residents? Can J Surg 2007 50 450 8 18053373 PMC2386217

[B27] Gilbert TB McGrath BJ Soberman M Chest tubes: indications, placement, management, and complications J Intensive Care Med 1993 8 73 86 10148363 10.1177/088506669300800203

